# The mental health status of offshore oil platform workers during the COVID-19 pandemic

**DOI:** 10.3389/fpsyt.2022.1009602

**Published:** 2022-10-11

**Authors:** Fereshteh Baygi, Nami Mohammadian Khonsari, Ehsan Seif, Hamid Asayesh, Mostafa Qorbani

**Affiliations:** ^1^Research Unit of General Practice, Department of Public Health, University of Southern Denmark, Odense, Denmark; ^2^Non-communicable Diseases Research Center, Alborz University of Medical Sciences, Karaj, Iran; ^3^Student Research Committee, Alborz University of Medical Sciences, Karaj, Iran; ^4^Department of Medical Emergencies, Qom University of Medical Sciences, Qom, Iran; ^5^Non-communicable Diseases Research Center, Endocrinology and Metabolism Population Sciences Institute, Tehran University of Medical Sciences, Tehran, Iran

**Keywords:** platform workers, offshore, mental health, COVID-19, PTSD, stress, anxiety, depression

## Abstract

**Background:**

Previous studies indicated that offshore workers have a high level of work-related stress on an everyday basis. This study aims to assess the prevalence and determinants of mental health conditions in offshore oil platform workers during the COVID-19 pandemic.

**Methods:**

Workers of three oil and gas platforms were assessed in this cross-sectional study. Their mental status was evaluated by the Posttraumatic Stress Disorder (PTSD-8) questionnaire, and Depression Anxiety, Stress Scales (DASS) questionnaires. Furthermore, we assessed satisfaction with life (SWL) with a single question. Finally, multivariate logistic regression was used to determine the association of demographic and work-related variables with mental health outcomes.

**Results:**

Overall, 278 (Males:197, Females: 81) out of 315 invited workers with a mean age of 35.6 (SD: 7.2) years were included in this study using a random sampling method (participation rate: 88.2%). PTSD symptoms and Insomnia were observed in 9 (3.2%) and 138 (49.6%) of the participants, respectively. The prevalence of stress, anxiety, and depressive symptoms were 72 (25.9%), 70 (24.6%), and 85 (30.5%), respectively. Based on multivariable (adjusted) logistic regression analysis, women had significantly higher odds of stress and anxiety than men; those with an academic education were significantly more dissatisfied with their lives than those without an academic education.

**Conclusion:**

Our findings revealed a high prevalence of anxiety, depressive symptoms, and stress among offshore oil platformers during the COVID-19 pandemic, especially in women. Indicating that women and those with a higher education level in the oil platform work settings are more susceptible to stressors.

## Background

The late COVID-19 has caused devastation worldwide in the past few years and has affected nearly all industries and working groups directly and indirectly (1, 2). The fear of exposure to the virus itself, economic and financial crises, and lifestyle changes are among the very few outcomes of the pandemic, with each one acting as a potential stressor affecting the population's mental status (1–4). Many studies evaluated the mental health of different occupations, especially health care workers (HCWs), who were under the most pressure and suffered the most adverse mental effect (4). Although other workers were far less exposed to the virus than HCWs, they suffered the effect of these many stressors to some extent (2, 5).

The oil and gas industry plays a crucial role in the economy and is one of the primary sources of employment in oil-producing countries (6). However, this occupation is affected by stressors from both the workplace and outside the workplace (7), which rests heavily on the workers' shoulders and affects their mental and physical health (7, 8). Previous studies indicate that offshore workers have a high level of work-related stress (8, 9). In addition, many studies have shown that the late COVID-19 pandemic resulted in massive adverse mental outcomes among the general population and different occupations (3–5, 8–11). In this regard, since the oil industry workers who are already affected by various stressors might be more susceptible to the adverse effects of the COVID-19 pandemic. However, no published studies assessed the mental status of these workers during the COVID-19 pandemic. This study aims to assess the prevalence and determinants of mental health conditions in offshore oil platform workers during the COVID-19 pandemic.

## Materials and methods

### Study design

This cross-sectional study was conducted between August and October 2021 among oil and gas platform workers in the Persian Gulf, Iran.

### Sample size and sampling method

The sample size was calculated according to a previous Seafarers' mental health study (12). Considering a prevalence of depression of 25%, an alpha error of 5%, and an attrition rate of 10%, the needed sample was estimated to be 315 participants. Participants were selected using a stratified random sampling method. First, five platforms were randomly selected from the 20 existing oil platforms. Then, participants were randomly selected, proportional to the size of the participants in each platform.

### Data collection

Data gathering was done through self-administrated online questionnaires. The original link was distributed among the workers through the platforms' headquarters. In addition, demographic variables and work-related characteristics (e.g., sex, age, marital status (single/married), work experience (in years), work status (day shift/night shift), and educational level (having an academic education/not having an academic education) were also gathered *via* online questionnaires.

### Instruments

Mental health status was assessed using valid standardized Persian questionnaires, including the Depression, Anxiety and Stress Scale (DASS-21) (13, 14), Posttraumatic Stress Disorder (PTSD-8) (15), and Insomnia severity index (ISI) (16, 17).

### DASS-21

DASS-21 is vastly used to measure depression, anxiety, and stress symptoms (13, 14). The reliability and validity of the Persian version of this questionnaire have been evaluated in studies with a satisfactory Cronbach's alpha of 0.94 for the total scales of the questionnaire (18). Each domain of anxiety, stress, and depression, consists of seven questions. In these questions, the answerer has four choices varying from zero (“It does not apply to me”) to three (“It applies to me completely”). The sum of the scores in each domain indicates the existence and the severity of the aforementioned conditions (14). Scores above seven, nine and fourteen indicate a high probability of depression, anxiety and stress in each subdomain, respectively (14).

### PTSD-8

PTSD is a short effective tool to evaluate the existence of the three conditions of intrusion, avoidance, and hyperarousal within PTSD. The PTSD-8 scale is comprised of eight questions. These questions are scored based on a four-point Likert scale (1 = not at all, 4 = very often) (15). Answering a question as “three” or “four” is considered the existence of the possible condition within the aforementioned domain and possible PTSD. This questionnaire was also validated in Persian with a satisfactory Cronbach's alpha of 0.86 (4).

### ISI

ISI is a short seven items tool to evaluate insomnia severity during 2 weeks prior to the test (16, 17). Its questions are scored based on a four-point Likert scale (0 = not at all, 4 = extreme). The total score, which is the sum of the scores of all items, falls within one of the domains of “no insomnia”, “subthreshold insomnia” (8 to 14), “moderate clinical insomnia” (15 to 21), and “severe clinical insomnia” (22 to 28) (17). the Persian version of this questionnaire has been validated with an acceptable Cronbach's alpha of (0.87) (19).

### Satisfaction with life (SWL) and perceived health

We assessed satisfaction with life and perceived health by using a single question regarding each variable. We asked the participants to rate their satisfaction with life (SWL) from one to 10 (1 = completely dissatisfied to 10 = completely satisfied), and rate their perception of their own health status from one to four (1 &2 = bad, 3 & 4 = good).

### Statistical analysis

We used Statistical Package for the Social Sciences Software, version 20 (SPSS) for data analyses. The Kolmogorov-Smirnov test evaluated the normal distribution of continuous variables. We expressed categorical variables as frequency and percentage and continuous variables as mean and standard deviation (SD). The prevalence of mental symptoms are reported alongside their 95% confidence interval (CI).

Man-Whitney U and Fisher's exact test tests were used to compare continuous and categorical variables within DASS, and PTSD subscales. In addition, the correlations between PTSD, DASS subscales and quantitative demographic variables were assessed using Spearman's correlation coefficient.

Univariable (crude) and multivariable (adjusted for variables with a *P*-value < 0.1 in the crude model) logistic regression analyses were performed, to determine the association between demographics and the aforementioned mental health conditions, and their results were reported as odds ratio (OR) and 95% CI. A *p*-value below 0.05 (two-tailed) was considered statistically significant.

## Results

Of the *315* invited workers, *278* completed the online questionnaires (participation rate: 88.2%). The mean age of participants was 35.42 ± 7.52 years. *197* (*84.2%*) of the participants were male, *67* (*24.1%*) were night shift workers, *210* (*75.5%*) were married, and *243 (87.4%)* had an academic education. The mean work experience was 11.44 ± 6.79 years. Among the participants, *73* (*26.2%*) were dissatisfied with their lives (Mean total SWL score: 6.74 ± 2.56). PTSD symptoms were observed in *9* (*3.2%*) of participants (Mean total PTSD score: 4.01 ± 3.92). The prevalence of stress, anxiety, and depressive symptoms (DASS 21 subscales) among participants were *72* (*25.9%*), *70* (*24.6%*), and *85* (*30.5%*), respectively. Regarding ISI, *138* (*49.6%*) participants had experienced some degree of insomnia (Mean total ISI score: *7.83* ± *3.79*).

The comparison of mental issues across demographic variables can be seen in Table 1. As shown no significant differences were seen in depression, anxiety, and PTSD subscales across age, work experience, marital status, and shift schedules. However, younger and less experienced participants endured significantly higher stress levels. Moreover, stress, anxiety and PTSD were significantly higher among women compared to men. Moreover, SWL and good health-perception was significantly higher in married participants and those with an academic education compared to the singles and those without an academic education.

**Table 1:** Comparison of mental issues across demographic variables.

**Variable**		**Age (Year)^a^**	***P*-value^M^**	**Sex** ^**b**^		***P*-value^F^**	**Marital status** ^**b**^		***P*-value^F^**	**Shift** ^**2**^		***P*-value^F^**	**Education** ^**b**^		***P*-value^F^**	**Work EXP (Year) ^1^**	***P*-value^M^**
				**Male**	**Female**		**Single**	**Married**		**Day**	**Night**		**Academic**	**Non Academic**			
Depression	No	35.6 ± 7.3	0.78	168 (84.8)	30 (15.2)	0.37	44 (22.7)	150 (77.3)	0.33	144 (77)	43 (23)	0.09	170 (85)	30 (15)	0.5	11.3 ± 6.7	0.81
	Yes	35.6 ± 6.6		66 (82.5)	14 (17.5)		21 (25.9)	60 (74.1)		51 (68)	24 (32)		73 (85.9)	12 (14.1)		11.4 ± 6.3	
Stress	No	36 ± 7.1	0.04^*^	183 (87.1)	27 (12.9)	0.02^*^	44 (21.2)	164 (78.8)	0.06	151 (76.3)	47 (23.7)	0.15	184 (86.4)	29 (13.6)	0.23	11.8 ± 6.6	0.02^*^
	Yes	34.6 ± 7		51 (75)	17 (25)		21 (31.3)	46 (68.7)		44 (68.8)	20 (31.3)		59 (81.9)	13 (18.1)		9.7 ± 6.3	
Anxiety	No	36.3 ± 7.4	0.04^*^	183 (87.6)	26 (12.4)	0.008^*^	50 (24.2)	157 (75.8)	0.43	152 (77.2)	45 (22.8)	0.06	181 (84.2)	34 (15.8)	0.24	11.8 ± 6.8	0.06
	Yes	33.7 ± 6		51 (73.9)	18 (26.1)		15 (22.1)	83 (77.9)		43 (66.2)	22 (33.8)		62 (88.6)	8 (11.4)		9.9 ± 5.8	
PTSD	No	35.6 ± 7.1	0.92	230 (85.5)	39 (14.5)	0.006^*^	63 (23.7)	203 (76.3)	0.64	187 (73.6)	67 (26.4)	0.09	235 (85.1)	41 (14.9)	0.61	10.5 ± 6.6	0.85
	Yes	35.2 ± 7.1		4 (44.4)	5 (55.6)		2 (22.2)	7 (77.8)		8 (100)	0		8 (88.9)	1 (11.1)		10.5 ± 6.4	
SWL	No	35.4 ± 8.5	0.15	60 (88.2)	8 (11.8)	0.19	25 (36.2)	44 (63.8)	0.004^*^	44 (67.7)	21 (32.3)	0.1	55 (75.3)	18 (24.7)	0.006^*^	10.4 ± 7.2	0.08
	Yes	35.7 ± 6.6		174 (82.9)	36 (17.1)		40 (19.4)	166 (80.6)		151 (76.6)	46 (23.4)		188 (88.7)	24 (11.3)		10.7 ± 6.4	
Perceived	Bad	34.15 ± 7.9	0.2	28 (80)	7 (20)	0.3	13 (38.2)	21 (61.8)	0.03^*^	21 (63.6)	12 (36.4)	0.1	29 (74.4)	10 (25.6)	0.04^*^	9.9 ± 5.5	0.23
health status	Good	35.8 ± 7		206 (84.8)	37 (15.2)		52 (21.6)	189 (78.4)		174 (76)	55 (24)		214 (87)	32 (13)		11.5 ± 6.7	
ISI	Normal	36.4 ± 7.5	0.44	127 (87)	19 (13)	0.12	32 (22.1)	113 (77.9)	0.31	112 (81.8)	25 (18.2)	0.003^*^	125 (85)	22 (15)	0.52	11.9 ± 6.8	0.08
	Insomnia	34.7 ± 6.6		107 (81.1)	25 (18.9)		33 (25.4)	97 (74.6)		83 (66.4)	42 (33.6)		118 (85.5)	20 (14.5)		10.6 ± 6.3	

a Are Reported As Mean (SD); b^2^Are Reported As N (%);

M Based On Mann-Whitney U Test;

F Based On Fisher's Exact test. Exp: Experience; SWL, Satisfaction With Life; ISI, Insomnia Severity Index; PTSD, Post-Traumatic Stress Disorder;

* Statistically Significant (P-value < 0.05).

The correlation coefficiencies (Table 2) show a strong negative correlation between SWL and DASS subscales (*p-value* < *0.05*). The same can be seen for SWL and PTSD subscales, and ISI scores as well (*p-value* < *0.05*). Furthermore, PTSD subscales and DASS subscales scores strongly correlate with one another.

**Table 2:** Spearman's correlation.

**Variable**	**Age**	**Work. Exp**	**SWL**	**DASS stress**	**DASS Anxiety**	**DASS depression**	**ISI score**	**PTSD score**
Age	1.000	0.897^**^	0.025	−0.081	−0.118	−0.071	−0.079	0.045
Work.Exp	0.897^**^	1.000	0.032	−0.101	−0.095	−0.067	−0.127*	0.079
SWL	0.025	0.032	1.000	−0.514^**^	−0.410^**^	−0.580^**^	−0.369^**^	−0.265^**^
DASS Stress	−0.081	−0.101	**–**0.0514^**^	1.000	0.720^**^	0.802^**^	0.570^**^	0.569^**^
DASS Anxiety	−0.118	−0.095	−0.410^**^	0.720^**^	1.000	0.666^**^	0.488^**^	0.573^**^
DASS Depression	−0.071	−0.067	−0.580^**^	0.802^**^	0.666^**^	1.000	0.581^**^	0.533^**^
ISI Score	−0.079	−0.127*	−0.369^**^	0.570^**^	0.488^**^	0.581^**^	1.000	0.386^**^
PTSD Score	0.045	0.079	−0.265^**^	0.569^**^	0.573^**^	0.533^**^	0.386^**^	1.000

** Statistically significant (P–value < 0.01); Exp, experience; SWL, Satisfaction With Life; DASS, the Depression Anxiety Stress Scale; ISI, Insomnia severity index; PTSD, Post–traumatic stress disorder.

The results of the logistic regression analysis are shown in Table 3. In the univariate (crude) logistic analysis, older age decreased the odds of anxiety by 5% [OR:0.95, 95%CI (0.91–0.99)], women compared to men had significantly higher odds of PTSD symptoms and stress by 637% [OR:7.37, 95%(1.89–28.66)] and 125% [OR:2.25, 95%CI (1.14–4.46)] respectively. Having more work experience decreased the odds of anxiety and stress by 5% [OR:0.95, 95%CI (0.91–0.99)] and 6%, (OR:0.94 95%CI (0.90–0.99)). Married participants had 125% higher odds of reporting having a bad self-health perception [OR:2.25, 95%CI (1.05–4.79)] and 135% higher odds of being dissatisfied with their lives [OR:2.35, 95%CI (1.29–4.29)] compared to single participants. Night shift workers had 126% higher odds of experiencing insomnia [OR:2.26, 95%CI (1.28–4.01)] compared to day shift workers. Those with an academic education had 130% higher odds of having a bad self-health perception [OR:2.30, 95%CI (1.02–5.17)] and 156% higher odds of being dissatisfied with their lives [OR:2.56, 95%CI (1.29–5.06)] compared to those without an academic education. In the multivariable (adjusted) logistic regression analysis, women had 229% higher odds of anxiety [OR:3.29, 95%CI (1.43–7.55)] and 180% higher odds of stress symptoms [OR:2.80, 95%CI (1.22–6.39)] compared to men. Night shift workers had 103% increased odds of experiencing insomnia [OR:2.03, *95%CI* (1.08–3.84)] compared to day shift workers. Those with an academic education had 293% higher odds of being dissatisfied with their lives [OR:3.93, 95%CI (1.57–9.86)].

Table 3Association of demographic and work-related variables with psychiatric symptoms and perceived health status in logistic regression analysis among seafarers.

**Table:** 

**Variable**		**PTSD (yes/no)**		**Anxiety (yes/no)**		**Stress (yes/no)**		**Depression (yes/no)**	
		**Model I**	**Model II**	**Model I**	**Model II**	**Model I**	**Model II**	**Model I**	**Model II**
Age (year)		0.99 (0.91–1.08)	1.08 (0.85–1.38)	0.95 (0.91–0.99)*	0.90 (0.80–1.01)	0.96 (0.93–1.00)	1.03 (0.93–1.14)	0.99 (0.96–1.03)	1.00 (0.91–1.11)
Sex (female/male)	Male	1	1	1	1	1	1	1	1
	Female	7.37 (1.89–28.66)*	14.08 (2.59–76.52)	2.48 (1.26–4.88)	3.29 (1.43–7.55)*	2.25 (1.14–4.46)*	2.80 (1.22–6.39)*	1.18 (0.59–2.38)	1.77 (0.78–4.02)
Work Exp (year)	0.97 (0.88–1.08)	0.98 (0.77–1.24)	0.95 (0.91–0.99)*	1.04 (0.92–1.17)	0.94 (0.90–0.99)*	0.92 (0.82–1.03)	0.99 (0.96–1.03)	0.99 (0.89–1.10)
Marital status	Single	1	1	1	1	1	1	1	1
	Married	1.08 (0.22–5.36)	1.61 (0.15–16.32)	1.12 (0.58–2.16)	1.89 (0.83–4.31)	0.58 (0.31–1.08)	0.71 (0.33–1.53)	1.03 (0.49–2.18)	0.96 (0.45–2.03)
Work status	Day	1	1	1	1	1	1	1	1
	Shift	N.A	N.A	1.72 (0.93–3.18)	1.30 (0.62–2.71)	1.46 (0.78–2.71)	1.44 (0.69-2.99)	1.62 (0.82-3.20)	0.61 (0.31-1.21)
Education	No	1	1	1	1	1	1	1	1
	Yes	1.39 (0.17–11.45)	N.A	1.45 (0.64–3.31)	0.70 (0.25–1.89)	0.71 (0.34–1.46)	0.49 (0.18–1.29)	0.96 (1.01–0.38)	1.01 (0.38–2.70)

**Table:** 

**Variable**		**Health status (bad/good)**		**SWL (dissatisfied/satisfied)**		**ISI (normal/insomnia)**	
		**Model I**	**Model II**	**Model I**	**Model II**	**Model I**	**Model II**
Age (year)		1.03 (0.98–1.08)	1.11 (0.96–1.29)	1.01 (0.97–1.05)	0.94 (0.84–1.04)	0.98 (0.95–1.01)	0.98 (0.89–1.07)
Sex (female/male)	Male	1	1	1	1	1	1
	Female	0.71 (0.29–1.76)	0.60 (0.20–1.77)	1.55 (0.68–3.52)	1.75 (0.61–5.04)	1.56 (0.81–2.99)	2.01 (0.91–4.45)
Work Exp (year)		1.04 (0.98–1.10)	0.90 (0.77–1.05)	1.03 (0.98–1.07)	1.07 (0.96–1.20)	0.96 (0.93–1.00)	1.00 (0.91–1.10)
Marital status	Single	1	1	1	1	1	1
	Married	2.25 (1.05–4.79)*	1.97 (0.78–4.79)	2.35 (1.29–4.29)*	2.07 (0.97–4.24)	0.83 (0.47–1.45)	0.94 (0.47–1.86)
Shift status (day/night)	Day	1	1	1	1	1	1
	Night	0.55 (0.25–1.19)	0.58 (0.23–1.44)	1.19 (0.42–3.33)	0.56 (0.27–1.15)	2.26 (1.28–4.01)*	2.03 (1.08–3.84)*
Education	No	1	1	1	1	1	1
	Yes	2.30 (1.02–5.17)*	2.32 (0.72–7.47)	2.56 (1.29–5.06)*	3.93 (1.57–9.86)*	1.03 (0.53–2.00)	0.85 (0.36–2.00)

Model I, Crude Mode; Model II, Adjusted for variables which had P-Value < 0.1 in the crude model values are reported as odds ratio (95% Confidence Interval); *Statistically significant (P-value < 0.05).

Exp, experience; SWL, Satisfaction With Life; ISI, Insomnia severity index; ISI, scores from 8 and above were considered as having insomnia.

## Discussion

There have been many studies on the mental health outcomes of the COVID-19 pandemic in different populations such as the general population (10), healthcare workers (3, 20), and seafarers (5). However, offshore oil platformers have not been well studied. Based on our findings, the prevalence of anxiety, depressive, and stress symptoms were 24.8, 28.8, and 24.4%, respectively. The prevalence of the aforementioned mental health problems among platform workers is very high compared to other occupations such as seafarers (5), health care workers, and other settings (4, 17). This can be due to other potential stressors that indirectly resulted from the pandemic (economic and financial difficulties, the induced fear of infection, etc.) alongside the work's nature itself. It should be noted that the prevalence of anxiety and depression among 1,747 offshore workers just prior to the beginning of the pandemic were 15 and 18 percent, respectively (21). Thus in the same field of work, the prevalence of these symptoms have increased about 10%. Although this difference in the prevalence of mental symptoms can be due to the difference in the countries in which these studies were conducted, one cannot deny the pandemic's effects on the increased prevalence of these adverse mental symptoms. Thus, the authors of the current study believe that individual strategies parallel with companies' mental health promotion policies should be developed in order to support workers in similar health emergencies in the future. Within these strategies, those who are at higher risk of being affected by stressors (such as women and those with a higher educational level) should be prioritized

Despite the high prevalence of stress among platformers workers, only 2.2% of them showed signs of PTSD. This prevalence is lower than the reported prevalence of PTSD symptoms among seafarers (37.3%) (22). Such a difference between our findings and seafarers might be due to the difference in the nature of the jobs (e.g., long time of social isolation among seafarers compared to platform workers) as well as the work setting itself (e.g., moving nature of the ships and related stressors compare to the fixed platforms).

Furthermore, our results indicate no significant relationship between marital status, shift schedule, and mental health after adjustment for confounding variables.

Nonetheless, those with a higher educational level had higher SWLs. This finding could be attributed to, higher income, and lower work-life conflicts (23).

Furthermore, the prevalence of stress and anxiety were significantly higher among females compared to the males in our study; this finding is in line with similar studies on male-dominated occupations (24). This difference of stress and anxiety among sexes can attributed to the different roles, responsibilities, concerns and stressors that female workers have (24, 25). In this regard it is suggested that in occupations dominated by one sex, additional psychological support is needed for the opposite sex. However, the low number of female participants in the current study might have adversely affected our findings. In order to evaluate the effect of such parameters on the mental health of the participants, we suggest a more extensive population-based study with a normal distribution of sex and marital status. Furthermore, conducting qualitative research to explore predictors and patterns of underlying processes of offshore workers' mental health status is recommended. It is well known that during this pandemic, the prevalence of mental disorders has risen; therefore, it is suggested that some psychosocial counseling interventions can be helpful (3, 4, 22, 26–28); and in times of global devastation, many workers will endure some degrees of mental distress, especially in occupations with high levels of stress. Hence, in dire times, psychological support should be prioritized for occupations with the most stress levels.

## Limitations and strengths of the study

Few studies have assessed offshore oil platform workers' stress and mental health status. Thus, due to the point above and the cross-sectional nature of the study, it was not possible to determine whether the found conditions were the pandemic's results or the workload. Furthermore, the self-report measurement might have affected the results. The participants were mostly male, this limitation might also affect the results of current study. Nonetheless, one strong point is that this study is one of the very few studies to evaluate the mental health status of offshore oil platform workers during the pandemic. The high response rate is due to the great follow up system and cooperation of the platforms' headquarters was another strong point.

## Conclusion

Our findings revealed a high prevalence of anxiety, depressive symptoms, and stress among offshore oil platformers during the COVID-19 pandemic. We urge all researchers to do more studies on these workers in order to cover all aspects of mental health status during COVID-19. Furthermore, the relevant authorities of the oil industry are encouraged to take proper action regarding workers' mental health issues in this setting.

## Data availability statement

The raw data supporting the conclusions of this article will be made available by the authors, without undue reservation upon request.

## Ethics statement

The study involving human participants was reviewed and approved by National Institute for Medical Research Development (NIMAD). The patients/participants provided their written informed consent to participate in this study.

## Author contributions

MQ and FB conceived the study, participated in study design, data collection, interpretation of the result, and revised the manuscript critically. NM participated in data collection, interpretation of the result, and wrote the first draft of the manuscript. HA and ES participated in data collection and drafting the manuscript. All authors contributed to the article and approved the submitted version.

## Funding

This study was funded by the National Institute for Medical Research Development (NIMAD) (Grant No. 4000207). The funder had no role in study design, data collection and analysis, decision to publish, or preparation of the manuscript.

## Conflict of interest

The authors declare that the research was conducted in the absence of any commercial or financial relationships that could be construed as a potential conflict of interest.

## Publisher's note

All claims expressed in this article are solely those of the authors and do not necessarily represent those of their affiliated organizations, or those of the publisher, the editors and the reviewers. Any product that may be evaluated in this article, or claim that may be made by its manufacturer, is not guaranteed or endorsed by the publisher.
